# Venous Thromboembolism in Denmark: Seasonality in Occurrence and Mortality

**DOI:** 10.1055/s-0039-1692399

**Published:** 2019-06-18

**Authors:** Nils Skajaa, Erzsébet Horváth-Puhó, Kasper Adelborg, Paolo Prandoni, Kenneth J. Rothman, Henrik Toft Sørensen

**Affiliations:** 1 Department of Clinical Epidemiology, Aarhus University Hospital, Aarhus, Denmark; 2 Department of Clinical Biochemistry, Aarhus University Hospital, Aarhus, Denmark; 3 Arianna Foundation on Anticoagulation, Bologna, Italy; 4 RTI Health Solutions, Research Triangle Institute, Research Triangle Park, North Carolina, United States; 5 Department of Epidemiology, Boston University School of Public Health, Boston, Massachusetts, United States

**Keywords:** seasonality, epidemiology, peak-to-trough ratio, venous thromboembolism

## Abstract

**Background** Many cardiovascular conditions exhibit seasonality in occurrence and mortality, but little is known about the seasonality of venous thromboembolism.

**Methods** Using Danish registries, we identified all patients with deep vein thrombosis, pulmonary embolism, splanchnic vein thrombosis, cerebral vein thrombosis, and retinal vein thrombosis during 1977–2016. We tallied monthly deaths occurring within 90 days of the venous thromboembolism diagnosis. We estimated peak-to-trough ratios and timing of the peak of both diagnoses and deaths summed over all years of the study period. The departure from 1.0 of the peak-to-trough ratio measures the intensity of any seasonal pattern.

**Results** We estimated a peak-to-trough ratio of 1.09 (95% confidence interval: 1.07–1.11) for deep vein thrombosis and 1.22 (1.19–1.24) for pulmonary embolism occurrence. The peak-to-trough ratios for splanchnic vein thrombosis, cerebral vein thrombosis, and retinal vein thrombosis occurrence were 1.10 (1.01–1.20), 1.19 (1.00–1.40), and 1.12 (1.07–1.17), respectively. The occurrence of all conditions peaked during winter or fall. In time trend analyses, the peak-to-trough ratio increased considerably for splanchnic vein thrombosis, cerebral vein thrombosis, and retinal vein thrombosis occurrence. In associated mortality, the peak-to-trough ratio for deep vein thrombosis was larger (1.15, 1.07–1.23) than that for pulmonary embolism (1.04, 1.01–1.08).

**Discussion** Excess winter risks were modest, but more marked for pulmonary embolism occurrence than for deep vein thrombosis occurrence. The seasonal pattern intensified throughout the study period for splanchnic vein thrombosis, cerebral vein thrombosis, and retinal vein thrombosis. The winter peak in mortality following pulmonary embolism was smaller than that for deep vein thrombosis.

## Introduction

Venous thromboembolism (VTE) typically manifests as deep vein thrombosis or pulmonary embolism, but affects any venous circulation. 1 Occurrences at other sites include splanchnic vein thrombosis (encompassing thrombosis of the portal, hepatic, mesenteric, and splenic veins) cerebral vein thrombosis, and retinal vein thrombosis. 2

VTE represents a growing global burden: the incidence rate is increasing due to changes in demographics, increases in the prevalence of several risk factors, and improvements in diagnostic imaging. 1 The risk of dying within 30 days following a VTE diagnosis is 5-, 80-, and 40-fold higher in patients suffering from deep vein thrombosis, pulmonary embolism, or splanchnic vein thrombosis compared with the general population. 3 4 The risks generally remain increased over the long term. 3 4

Occurrence of cardiovascular diseases is unequally distributed throughout the year, as is associated mortality. 5 6 But most evidence regarding seasonality relates to atherosclerotic disease rather than to VTE. 6 The existing literature on the seasonality of VTE is conflicting, with some studies reporting excess risk during the winter, 7 8 9 10 11 12 13 14 and others reporting no such effect. 15 16 17 Most previous studies focused exclusively on deep vein thrombosis and pulmonary embolism. 7 8 9 10 11 12 13 14 15 16 17 Studies on cerebral vein thrombosis seasonality have been small (<200 patients) and inconclusive, 18 19 20 while, to the best of our knowledge, no previous study has investigated the seasonality of splanchnic vein thrombosis and retinal vein thrombosis. As improved imaging modalities lead to more accurate diagnoses, it may be time to revisit earlier findings. 7 15 16

Several biological mechanisms could contribute to the seasonality of VTE. 6 Seasonal alterations in ambient temperature are considered pivotal, as exposure to cold promotes acute and chronic physiological changes, including elevations in both peripheral vasoconstriction, sympathetic nervous system activity, blood viscosity, fibrinogen levels, and C-reactive protein levels—in turn, these changes may trigger adverse cardiovascular events. 6 8 Acute infections, occurring most often in winter, also are associated with increased risk of deep vein thrombosis and pulmonary embolism. 21 Moreover, the seasonal pattern likely depends on the susceptibility of individual patients. Thus, deep vein thrombosis or pulmonary embolism in patients with a recent immobilization due to a medical condition or surgery may follow a different seasonal pattern than in those without such provoking factors.

To add to the understanding of the seasonal pattern of VTE, we undertook a nationwide population-based study in Denmark, using data from 1977 to 2016.

## Methods

### Setting

This study was based on data obtained from Danish healthcare and administrative registries. The Danish healthcare system is government-funded, ensuring free access to health care for all legal residents. 22 The unique 10-digit identifier assigned to all residents at birth or upon immigration by the Danish Civil Registration System (CRS) allows complete individual-level linkage of all health and administrative registries. 23 The Danish National Patient Registry (DNPR) contains data on more than 99% of all discharges from Danish hospitals. 24 Each hospital discharge (available from 1977) or outpatient visit (available from 1995) is recorded in the DNPR with one primary diagnosis and one or more secondary diagnoses coded according to the *Eighth Revision of the International Classification of Diseases* (ICD) during 1977–1993 and according to the *Tenth Revision* thereafter. 24

### Patients with Venous Thromboembolism

We searched the DNPR and identified all inpatients and outpatients with a first-time primary or secondary discharge diagnosis of deep vein thrombosis, pulmonary embolism, splanchnic vein thrombosis (January 1, 1977, through December 31, 2016), cerebral vein thrombosis, and retinal vein thrombosis (1 January 1994 through 31 December 2016) based on ICD diagnosis codes. 24 Due to the paucity of cerebral vein thrombosis and retinal vein thrombosis events before 1994 and the presumed low validity of ICD-8 diagnosis codes, we included only patients with an ICD-10 diagnosis code for these conditions. If a patient had a simultaneous diagnosis of pulmonary embolism and deep vein thrombosis, we used the pulmonary embolism diagnosis, owing to its higher mortality rate. 3 Patients with a diagnosis of splanchnic vein thrombosis, cerebral vein thrombosis, or retinal vein thrombosis and a concurrent diagnosis of any of the other conditions under study were considered in both analyses.

We also categorized a VTE as provoked or unprovoked. Patients with a preexisting cancer diagnosis as well as a fracture or trauma, surgery, pregnancy, or prolonged immobilization due to hospitalization within 90 days before a deep vein thrombosis or pulmonary embolism diagnosis were classified as having a provoked VTE, while those without these factors were classified as having an unprovoked VTE. 25 We defined prolonged immobilization as an inpatient stay of at least 14 days from the date of admission to discharge. 26 We considered an admission occurring on the same day as discharge from another admission a transfer between hospital departments rather than two separate admissions.

### Statistical Analysis

To assess seasonality, we summed monthly frequencies of each year in the study period. To adjust for varying length of month, we multiplied each monthly frequency by 30 and divided the result by the length of the month. We then applied Edwards' model, which assumes that the expected values for monthly frequencies follow a sine curve with a single annual cycle. 27 Based on this model, we estimated the peak-to-trough ratio of the summarized monthly frequencies using a sine curve fitted to the 12 adjusted monthly frequencies. 28 Edwards' model is sensitive to cyclic trends, and considerably more so than alternatives that do not involve fitting a cyclic curve to the data, for example, comparisons of discrete time periods. 27 The magnitude of the peak-to-trough ratio indicates the intensity of seasonality, equivalent to a risk ratio that contrasts risks for the peak month versus the trough month. 29 Unlike some other effect measures, the peak-to-trough ratio cannot take a value less than 1.0.

Because occurrence of VTE and associated mortality may follow different seasonal patterns, we searched the CRS to ascertain 90-day mortality counts following a diagnosis of deep vein thrombosis and pulmonary embolism (separately for provoked and unprovoked events), splanchnic vein thrombosis, cerebral vein thrombosis, and retinal vein thrombosis. We then estimated the peak-to-trough ratio in mortality by summarizing these mortality counts during the study period.

### Bias Assessment

Because the peak-to-trough ratio is always 1.0 or greater, variability of monthly frequencies even in the absence of any seasonality will produce estimates greater than 1.0. 30 Thus, as a bias analysis, we performed a plasmode simulation to assess the effect of randomness in the data. 31 We reassigned the summarized monthly frequencies at random and then estimated the peak-to-trough ratio. We repeated this process 1,000 times and computed the average of all simulations.

### Additional Analyses

To explore whether any biological interactions or cohort effects were present, we stratified the analysis of seasonality in occurrence of VTEs by sex, age group (0–29, 30–49, 50–69, and 70+ years), comorbidity based on Charlson Comorbidity Index scores (0, 1, 2, 3 + ), 32 calendar period (1977–1993, 1994–2008, and 2009–2016), type of diagnosis (primary, secondary), and department (inpatient, outpatient).

As a sensitivity analysis, we repeated the seasonal analysis of monthly frequencies of occurrence using a log-linear Poisson periodic regression model that allowed inclusion of covariates and secular trends in seasonality: log(disease occurrence) = seasonality + covariates + secular trend. 29

We used R, version 3.3.3, and SAS, version 9.4 (SAS Institute Inc., Cary, North Carolina) to conduct the analyses. The ICD codes used in the study are listed in Supplementary Tables S1 and S2 .

**Table 1:** Characteristics (no., %) of patients with deep vein thrombosis, pulmonary embolism, provoked VTE, unprovoked VTE, and splanchnic vein thrombosis during 1977–2016, and cerebral vein thrombosis and retinal vein thrombosis during 1994–2016

	Deep venous thrombosis	Pulmonary embolism	Provoked VTE	Unprovoked VTE	Splanchnic venous thrombosis	Cerebral venous thrombosis	Retinal venous thrombosis
All patients	101,895	84,080	69,908	116,067	3,972	1,118	15,706
Women	52,517 (51.5)	44,853 (53.3)	38,411 (54.9)	58,959 (50.8)	1,919 (48.3)	679 (61.0)	7,973 (50.8)
Median age (25th–75th percentiles), y	65 (51–76)	71 (60–79)	70 (59–79)	68 (52–77)	66 (53–77)	45 (27–62)	71 (62–79)
Age groups, y							
0–29	5,228 (5.1)	2,419 (2.9)	2,175 (3.1)	5,472 (4.7)	195 (4.9)	334 (29.9)	114 (0.7)
30–49	18,862 (18.5)	8,874 (10.6)	7,836 (11.2)	19,900 (17.1)	617 (15.5)	309 (27.6)	1,125 (7.2)
50–69	37,518 (36.8)	27,969 (33.3)	24,524 (35.1)	40,963 (35.3)	1,566 (39.4)	309 (27.6)	5,992 (38.2)
70+	40,287 (39.5)	44,818 (53.3)	35,373 (50.6)	49,732 (42.8)	1,594 (40.1)	166 (14.8)	8,475 (54.0)
Charlson Comorbidity Index score							
0	59,949 (58.8)	42,300 (50.3)	24,021 (34.4)	78,228 (67.4)	1,568 (39.5)	701 (62.7)	8,752 (55.7)
1	16,022 (15.7)	14,397 (17.1)	8,685 (12.4)	21,734 (18.7)	733 (18.5)	216 (19.3)	2,867 (18.3)
2	13,325 (13.1)	13,070 (15.5)	17,254 (24.7)	9,141 (7.9)	582 (14.7)	104 (9.3)	2,096 (13.3)
3+	12,599 (12.4)	14,313 (17.0)	19,948 (28.5)	6,964 (6.0)	1,089 (27.4)	97 (8.7)	1,991 (12.7)
Calendar period							
1977–1993	29,103 (28.6)	31,284 (37.2)	24,347 (34.8)	36,040 (31.1)	1,374 (34.6)	NA	NA
1994–2008	43,725 (42.9)	25,400 (30.2)	23,070 (33.0)	46,055 (39.7)	1,062 (26.7)	581 (52.0)	8,181 (52.1)
2009–2016	29,067 (28.5)	27,396 (32.6)	22,491 (32.2)	33,972 (29.3)	1,536 (38.7)	537 (48.0)	7,525 (47.9)
Type of diagnosis							
Primary	79,529 (78.0)	56,353 (67.0)	45,504 (65.1)	90,378 (77.9)	2,320 (58.4)	886 (79.2)	12,385 (78.9)
Secondary	22,366 (22.0)	27,727 (33.0)	24,404 (34.9)	25,689 (22.1)	1,652 (41.6)	232 (20.8)	3,321 (21.1)
Department							
Inpatient	86,920 (85.3)	79,370 (94.4)	63,691 (91.1)	102,599 (88.4)	3,551 (89.4)	976 (87.3)	590 (3.8)
Outpatient	14,975 (14.7)	4,710 (5.6)	6,217 (8.9)	13,468 (11.6)	421 (10.6)	142 (12.7)	15,116 (96.2)

Abbreviation: VTE, venous thromboembolism (deep venous thrombosis and pulmonary embolism).

**Table 2:** Peak-to-trough ratios (95% confidence intervals) of summarized monthly cases during 1977–2016 (deep vein thrombosis, pulmonary embolism, provoked VTE, unprovoked VTE, and splanchnic vein thrombosis) and during 1994–2016 (cerebral vein thrombosis and retinal vein thrombosis)

	Deep venous thrombosis	Pulmonary embolism	Provoked VTE	Unprovoked VTE	Splanchnic venous thrombosis	Cerebral venous thrombosis	Retinal venous thrombosis
All patients	1.09 (1.07–1.11)	1.22 (1.19–1.24)	1.15 (1.13–1.18)	1.13 (1.11–1.15)	1.10 (1.01–1.20)	1.19 (1.00–1.40)	1.12 (1.07–1.17)
Women	1.06 (1.03–1.09)	1.23 (1.20–1.27)	1.15 (1.11–1.18)	1.11 (1.08–1.14)	1.30 (1.10–1.53)	1.11 (1.00–1.38)	1.08 (1.01–1.15)
Men	1.13 (1.10–1.16)	1.20 (1.17–1.23)	1.16 (1.12–1.19)	1.16 (1.13–1.19)	1.17 (1.00–1.35)	1.12 (1.00–1.47)	1.15 (1.08–1.23)
Age groups, y							
0–29	1.05 (1.00–1.14)	1.25 (1.11–1.40)	1.08 (1.00–1.22)	1.11 (1.03–1.19)	1.09 (1.00–1.75)	1.23 (1.00–1.67)	1.06 (1.00–1.78)
30–49	1.05 (1.01–1.10)	1.06 (1.00–1.13)	1.09 (1.02–1.16)	1.08 (1.04–1.12)	1.24 (1.00–1.60)	1.09 (1.00–1.50)	1.20 (1.01–1.42)
50–69	1.10 (1.07–1.13)	1.23 (1.19–1.27)	1.16 (1.12–1.21)	1.14 (1.11–1.18)	1.30 (1.09–1.54)	1.10 (1.00–1.52)	1.12 (1.04–1.20)
70+	1.17 (1.13–1.20)	1.25 (1.22–1.29)	1.17 (1.13–1.20)	1.23 (1.20–1.26)	1.04 (1.00–1.26)	1.11 (1.00–1.71)	1.10 (1.03–1.17)
Charlson Comorbidity Index score							
0	1.13 (1.11–1.16)	1.25 (1.21–1.28)	1.22 (1.18–1.27)	1.16 (1.14–1.18)	1.10 (1.00–1.32)	1.16 (1.00–1.44)	1.09 (1.03–1.16)
1	1.08 (1.03–1.13)	1.20 (1.14–1.26)	1.11 (1.04–1.18)	1.11 (1.07–1.15)	1.27 (1.00–1.67)	1.19 (1.00–1.74)	1.16 (1.05–1.29)
2	1.03 (1.00–1.08)	1.17 (1.11–1.23)	1.12 (1.08–1.17)	1.05 (1.00–1.12)	1.01 (1.00–1.34)	1.07 (1.00–1.85)	1.07 (1.00–1.21)
3+	1.01 (1.00–1.06)	1.19 (1.14–1.25)	1.11 (1.06–1.15)	1.06 (1.00–1.14)	1.34 (1.01–1.63)	1.03 (1.00–1.82)	1.17 (1.03–1.33)
Calendar period							
1977–1993	1.17 (1.13–1.21)	1.23 (1.20–1.28)	1.18 (1.14–1.22)	1.22 (1.18–1.26)	1.04 (1.00–1.21)	NA	NA
1994–2008	1.07 (1.04–1.11)	1.19 (1.15–1.24)	1.13 (1.09–1.17)	1.10 (1.07–1.13)	1.15 (1.00–1.37)	1.08 (1.00–1.36)	1.07 (1.01–1.14)
2009–2016	1.04 (1.01–1.08)	1.22 (1.18–1.26)	1.14 (1.10–1.18)	1.09 (1.05–1.12)	1.20 (1.04–1.39)	1.29 (1.01–1.65)	1.16 (1.08–1.23)
Type of diagnosis							
Primary	1.09 (1.07–1.11)	1.22 (1.20–1.25)	1.15 (1.12–1.18)	1.13 (1.11–1.15)	1.12 (1.00–1.26)	1.10 (1.00–1.32)	1.11 (1.06–1.17)
Secondary	1.10 (1.06–1.15)	1.20 (1.16–1.24)	1.15 (1.11–1.19)	1.15 (1.11–1.19)	1.03 (1.00–1.18)	1.09 (1.00–1.57)	1.12 (1.02–1.24)
Department							
Inpatient	1.10 (1.08–1.12)	1.22 (1.19–1.24)	1.15 (1.13–1.18)	1.14 (1.12–1.26)	1.04 (1.00–1.15)	1.20 (1.00–1.43)	1.05 (1.00–1.33)
Outpatient	1.08 (1.03–1.13)	1.23 (1.13–1.33)	1.18 (1.10–1.27)	1.09 (1.04–1.15)	1.23 (1.00–1.62)	1.06 (1.00–1.70)	1.11 (1.06–1.16)
Plasmode simulation a	1.05 (1.03–1.07)	1.09 (1.07–1.11)	1.06 (1.04–1.09)	1.06 (1.05–1.09)	1.05 (1.00–1.14)	1.13 (1.00–1.31)	1.07 (1.03–1.12)

Abbreviations: NA, not applicable; VTE, venous thromboembolism (deep venous thrombosis and pulmonary embolism).

a Mean peak-to-trough ratio from random reassignment of monthly cases 1000 times.

### Ethics

According to Danish legislation, informed consent and approval from an ethics committee are not required for registry-based studies. The study was approved by the Danish Data Protection Agency (2015-57-0002).

## Results

We identified 101,895 patients with a first-time diagnosis of deep vein thrombosis, 84,080 with pulmonary embolism (of which 6,798 [8.1%] also had a concurrent diagnosis of deep vein thrombosis), 3,972 with splanchnic vein thrombosis, 1,118 with cerebral vein thrombosis, and 15,706 with retinal vein thrombosis. Among patients with deep vein thrombosis or pulmonary embolism, 116,067 (62%) events were unprovoked. The median age of patients with pulmonary embolism and retinal vein thrombosis (71 years) was higher than for those with deep vein thrombosis (65 years), splanchnic vein thrombosis (66 years), and cerebral vein thrombosis (45 years). The majority of cerebral vein thrombosis patients were women (61%; Table 1 , Supplementary Table S3 ).

**Table 3:** Peak-to-trough ratios (95% confidence intervals) of summarized monthly deaths during 1977–2016 (within 90 days following deep vein thrombosis, pulmonary embolism, provoked VTE, unprovoked VTE, and splanchnic vein thrombosis) and during 1994–2016 (within 90 days following cerebral vein thrombosis and retinal vein thrombosis)

	Deep venous thrombosis	Pulmonary embolism	Provoked VTE	Unprovoked VTE	Splanchnic venous thrombosis	Cerebral venous thrombosis	Retinal venous thrombosis
All patients	1.15 (1.07–1.23)	1.04 (1.01–1.08)	1.05 (1.01–1.09)	1.22 (1.17–1.28)	1.08 (1.00–1.24)	1.31 (1.00–2.52)	1.05 (1.00–1.74)
Plasmode simulation a	1.06 (1.00–1.13)	1.04 (1.01–1.08)	1.03 (1.00–1.07)	1.10 (1.06–1.14)	1.09 (1.00–1.23)	1.14 (1.00–2.01)	1.04 (1.00–1.68)

Abbreviation: VTE, venous thromboembolism (deep venous thrombosis and pulmonary embolism).

a Mean peak-to-trough ratio from random reassignment of monthly deaths 1,000 times.

### Seasonality in Occurrence

**Fig. 1:**
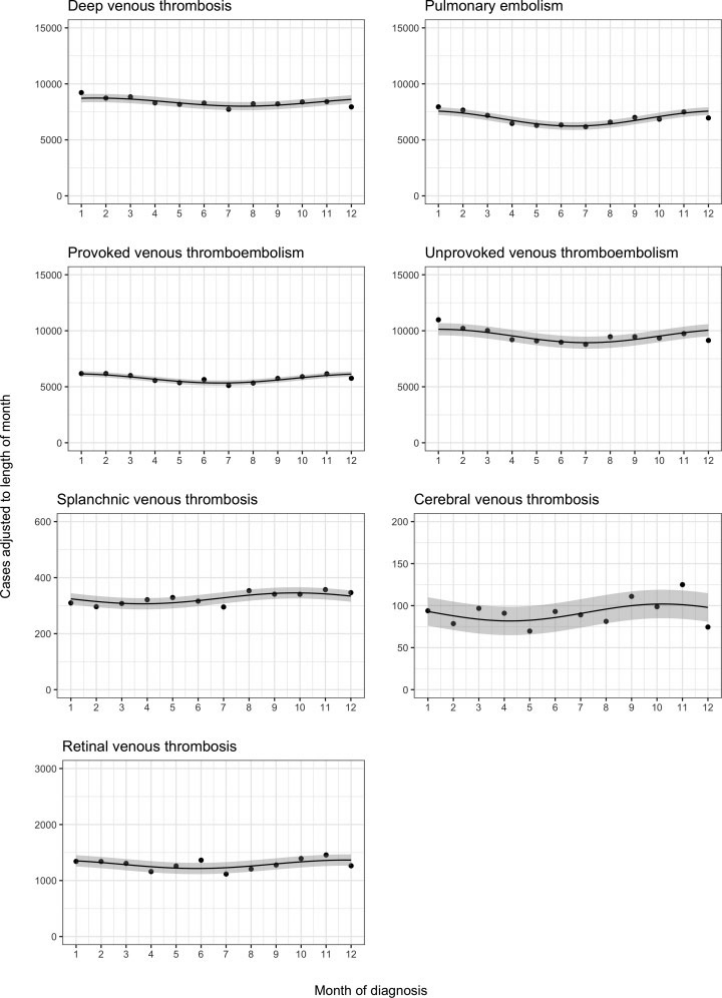
Summarized cases within each calendar month during 1977–2016 (occurrence of deep vein thrombosis, pulmonary embolism, provoked venous thromboembolism, unprovoked venous thromboembolism, splanchnic vein thrombosis) and 1994–2016 (occurrence of cerebral vein thrombosis and retinal vein thrombosis), adjusted for the length of month with a fitted sine curve and 95% confidence band.

Fig. 1 shows summarized monthly frequencies of occurrence during the entire study period with a fitted sine curve ( Supplementary Table S4 ). Overall, there was evidence of modest seasonal fluctuations: The peak-to-trough ratios of the summaries were 1.09 (95% confidence interval [CI]: 1.07–1.11) for deep vein thrombosis, 1.22 (95% CI: 1.19–1.24) for pulmonary embolism, 1.15 (95% CI: 1.13–1.18) for provoked VTE, 1.13 (95% CI: 1.11–1.15) for unprovoked VTE, 1.10 (95% CI: 1.01–1.20) for splanchnic vein thrombosis, 1.19 (95% CI: 1.00–1.40) for cerebral vein thrombosis, and 1.12 (95% CI: 1.07–1.17) for retinal vein thrombosis ( Table 2 ). The estimated day of peak occurrence was February 5 for deep vein thrombosis, January 2 for pulmonary embolism, January 9 for provoked VTE, January 17 for unprovoked VTE, October 22 for splanchnic vein thrombosis, October 25 for cerebral vein thrombosis, and December 11 for retinal vein thrombosis.

### Seasonality in 90-Day Mortality

**Fig. 2:**
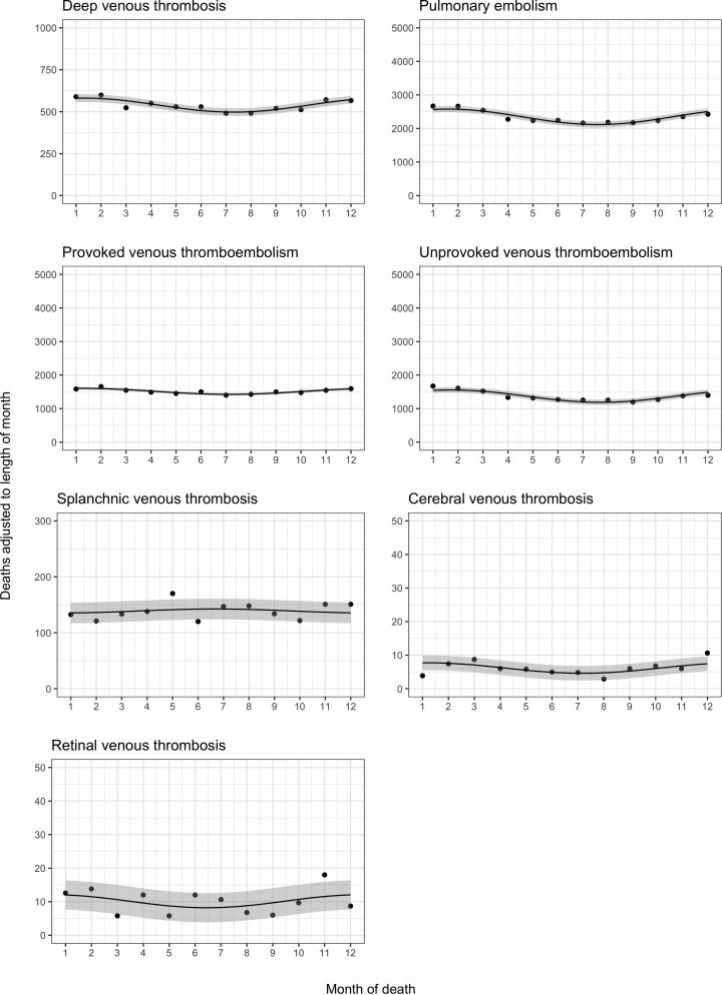
Summarized deaths within each calendar month during 1977–2016 (within 90 days following deep vein thrombosis, pulmonary embolism, provoked venous thromboembolism, unprovoked venous thromboembolism, and splanchnic vein thrombosis) and 1994–2016 (within 90 days following cerebral vein thrombosis and retinal vein thrombosis), adjusted for the length of month with a fitted sine curve and 95% confidence band.

During the entire study period, we identified 6,560 deaths within 90 days following a deep vein thrombosis, 28,560 deaths following a pulmonary embolism, 18,429 deaths following a provoked VTE, 16,691 deaths following an unprovoked VTE, 1,695 deaths following a splanchnic vein thrombosis, 75 deaths following a cerebral vein thrombosis, and 123 deaths following a retinal vein thrombosis ( Supplementary Table S5 ). Fig. 2 displays these monthly frequencies ( Supplementary Table S6 ). Again, the seasonal fluctuations in mortality were modest, although some were stronger than the patterns for VTE occurrence. For deep vein thrombosis, the peak-to-trough ratio was 1.15 (95% CI: 1.07–1.23); for pulmonary embolism, 1.04 (95% CI: 1.01–1.08); for provoked VTE, 1.05 (95% CI: 1.01–1.09); for unprovoked VTE, 1.22 (95% CI: 1.17–1.28); for splanchnic vein thrombosis, 1.08 (95% CI: 1.00–1.24); for cerebral vein thrombosis, 1.31 (95% CI: 1.00–2.52); and for retinal vein thrombosis, 1.05 (95% CI: 1.00–1.74) ( Table 3 ). The time of peak mortality was January 4 for deep vein thrombosis, February 1 for pulmonary embolism, December 2 for provoked VTE, January 29 for unprovoked VTE, June 10 for splanchnic vein thrombosis, March 23 for cerebral vein thrombosis, and November 6 for retinal vein thrombosis.

### Bias Assessment

After randomly reassigning the summarized monthly frequencies in VTE occurrence, the simulated mean peak-to-trough ratio for the entire study period was 1.05 (95% CI: 1.03–1.17) for deep vein thrombosis, 1.09 (95% CI: 1.07–1.11) for pulmonary embolism, 1.06 (95% CI: 1.04–1.09) for provoked VTE, 1.06 (95% CI: 1.06–1.09) for unprovoked VTE, 1.05 (95% CI: 1.00–1.14) for splanchnic vein thrombosis, 1.13 (95% CI: 1.00–1.31) for cerebral vein thrombosis, and 1.07 (95% CI: 1.03–1.12) for retinal vein thrombosis ( Table 2 ).

Similarly, we randomly reassigned summarized monthly mortality counts. The mean simulated peak-to-trough ratio was 1.06 (95% CI: 1.00–1.13) for deep vein thrombosis, 1.04 (95% CI: 1.01–1.08) for pulmonary embolism, 1.03 (95% CI: 1.00–1.07) for provoked VTE, 1.10 (95% CI: 1.06–1.14) for unprovoked VTE, 1.09 (95% CI: 1.00–1.23) for splanchnic vein thrombosis, 1.14 (95% CI: 1.00–2.01) for cerebral vein thrombosis, and 1.04 (95% CI: 1.00–1.68) for retinal vein thrombosis ( Table 3 ).

### Additional Analyses

In subgroups of patients with a VTE diagnosis, the peak-to-trough ratio was comparable for men and women for all VTE types. For deep vein thrombosis, provoked VTE, and unprovoked VTE, the peak-to-trough ratio largely increased with age while it decreased with higher Charlson Comorbidity Index scores. Conversely, for pulmonary embolism, splanchnic vein thrombosis, cerebral vein thrombosis, and retinal vein thrombosis, the peak-to-trough ratio did not fluctuate in any clear direction in these subgroups. Between 1977–1993 and 2009–2016, the peak-to-trough ratio decreased for deep vein thrombosis, but remained stable for pulmonary embolism. For splanchnic vein thrombosis, the peak-to-trough ratio increased considerably between calendar periods. A similar increasing trend was observed for cerebral vein thrombosis and retinal vein thrombosis between calendar periods. The peak-to-trough ratios were largely similar between primary/secondary and inpatient/outpatient diagnoses ( Table 2 ).

When we repeated the seasonal analyses with a log-linear Poisson regression model, peak-to-trough ratios were similar ( Supplementary Table S7 ).

## Discussion

Our summary data for the 1977–2016 period showed that occurrence of VTE follows a gentle seasonal pattern with a peak during winter, but with substantially different excess risks in winter for deep vein thrombosis (9%) and pulmonary embolism (22%). Seasonal risks were similar for provoked and unprovoked VTE. For splanchnic vein thrombosis, cerebral vein thrombosis, and retinal vein thrombosis, the excess winter risk was negligible during the first part of the study period and then increased. Excess winter risks in mortality were higher following deep vein thrombosis (15%) than following pulmonary embolism (4%).

Our findings are consistent in part with those of a 2011 meta-analysis of 12 studies including 23,469 patients with either deep vein thrombosis or pulmonary embolism. The meta-analysis reported a higher occurrence of VTE during winter, with a relative risk of 1.14 (99% CI: 1.14–1.15). 8 However, most patients ( *n* = 19,245, 82%) came from an Italian study 9 that found excess risks in winter based on hospital admissions for pulmonary embolism in the Emilia Romagna region. It did not include a large American study 16 that showed little seasonality over a 21-year summary period based on hospital discharges in the National Hospital Discharge Survey. Most, 10 11 12 13 14 but not all, 17 subsequent studies found similar findings to those from the meta-analyses. Of note, a Danish study of 152,548 patients above 20 years of age with a first-time discharge diagnosis of deep vein thrombosis or pulmonary embolism in the DNPR during 1980–2010 estimated a peak-to-trough ratio of 1.19 with a peak during winter. 12

Our results support previous research suggesting that seasonality of VTE increases at older ages. 11 14 Elderly patients have enhanced susceptibility to VTE due to respiratory tract infections and the associated inflammatory state. 14 However, this relation is inconsistent with our finding that the peak-to-trough ratio increased more markedly with age for unprovoked VTE compared with provoked VTE. Thus, the cause of the apparent age dependence is not known.

In contrast to previous studies, we conducted a simulation analysis allowing us to assess the degree of a seasonal pattern that chance might produce. Since the peak-to-trough ratio, a common estimator of seasonality, is biased upward, 30 earlier research using this estimator may have overestimated the seasonal intensity. 8 11 12 After taking into consideration the results from our simulation analysis, we conclude that any excess winter risks of deep vein thrombosis, splanchnic vein thrombosis, cerebral vein thrombosis, and retinal vein thrombosis occurrence are modest at best. In contrast, the excess risks of pulmonary embolism and provoked/unprovoked VTE occurrence were marked enough to persist even after subtracting the effect of the estimation bias. Thus, our findings demonstrate that deep vein thrombosis and pulmonary embolism occurrence follow distinct seasonal patterns. It is possible that presentation with an acute respiratory tract infection increased the probability of pulmonary embolism detection during the winter. We believe, however, that this is an insufficient explanation for the divergent seasonal risks between deep vein thrombosis and pulmonary embolism. Moreover, our findings indicate that the timing of peak pulmonary embolism occurrence precedes that of deep vein thrombosis occurrence. However, considering the uncertainty in estimating the timing of these peaks, both peaks should be interpreted more broadly as winter peaks.

We found comparable peak-to-trough ratios for provoked and unprovoked VTE occurrence, suggesting that none of the provoking factors plays a substantial seasonal role. Although the summary peak-to-trough ratios of splanchnic vein thrombosis, cerebral vein thrombosis, and retinal vein thrombosis were minimal after consideration of bias, we found a considerable secular trend for these conditions. Considering temporal improvements in diagnostic imaging, the estimated peak-to-trough ratios found in the later part of the study period may depict more accurately the seasonal pattern of these conditions.

We also examined the seasonality of monthly mortality counts. Previous studies on seasonality of VTE mortality have focused on pulmonary embolism, with most reporting peaks in winter. 33 Unexpectedly, in our study, the seasonal risks for deep vein thrombosis and pulmonary embolism mortality differed considerably, both between conditions and compared with the seasonal risks in occurrence. Indeed, the seasonal risks found for pulmonary embolism mortality was negligible when considering bias, in contrast to previous findings. 33 Similarly, we found a substantial difference in seasonality between provoked VTE and unprovoked VTE mortality despite similar seasonal risks in occurrence.

Ambient environmental conditions largely drive the seasonality of cardiovascular disease, including any observed seasonal pattern in occurrence of VTE and associated mortality. 6 However, the precise mechanisms underlying this driving force are multifactorial and unclear. Our findings suggest that any acute and chronic physiological changes in response to ambient temperature lead to a clear seasonal pattern in occurrence of pulmonary embolism but not in occurrence of deep vein thrombosis. Respiratory tract infections may be involved in the pathogenesis of VTE, particularly pulmonary embolism, due to local impairment of the coagulation cascade and associated systemic inflammation. Such infections may constitute a link to any seasonal pattern in occurrence. 21

Our estimates of seasonality in VTE occurrence are contingent on the validity of diagnoses in the DNPR. Of note, a validation study found positive predictive values of 86 and 90% for diagnoses of first-time deep vein thrombosis and pulmonary embolism. 34 The diagnosis of retinal vein thrombosis in the DNPR is assumed to have high validity because almost all patients are diagnosed at ophthalmologic departments. 35 While the splanchnic vein thrombosis and cerebral vein thrombosis diagnoses in the DNPR have not yet been validated, most splanchnic vein thrombosis diagnoses are based on an ultrasound examination or a computed tomography scan, 36 and it is unlikely that the validity of the cerebral vein thrombosis diagnosis diverges greatly from that of other VTE diagnoses.

In this population-based study covering 40 years, we found that VTE occurrence follows a seasonal pattern with a peak during winter. However, excess winter risks were marked for pulmonary embolism occurrence and less so for deep vein thrombosis occurrence. In contrast, we found pronounced seasonal risks in mortality within 90 days of a deep vein thrombosis but negligible risks following a pulmonary embolism. For splanchnic vein thrombosis, cerebral vein thrombosis, and retinal vein thrombosis occurrence, seasonal risks increased steadily over the study period.
