# Supplementary material for: Venous Thromboembolism in Denmark: Seasonality in Occurrence and Mortality
Source: TH Open. 2019 Jun 18;3(2):e171–9. doi: 10.1055/s-0039-1692399 (PMC6598086; doi:10.1055/s-0039-1692399)
Supplement: Supplementary file 1 — Supplementary Material [file 10-1055-s-0039-1692399-s190019.pdf]

Supplementary Material

Table S1 *International Classification of Diseases* (ICD) codes used in the study

	ICD-8	ICD-10	Other codes in the Danish National Patient Registry
Deep venous thrombosis	45100	I801-3	
Pulmonary embolism	45099	I26	
Splanchnic venous thrombosis			
Portal venous thrombosis	45299	I81.9	
Mesenteric venous thrombosis	44429	K55.0H	
Splenic venous thrombosis	28944, 45303	NA	
Hepatic venous thrombosis	45301	I82.0	
Cerebral venous thrombosis	NA	DI636, DI676	
Retinal venous thrombosis	37708	DH348	
Classic provoking factors			
Cancer, any previous diagnosis	140-209, 28710	C00-C99, D45, D473	
Fracture/trauma within previous 90 days	800-929, 950-959	S00-T14	
Surgery within previous 90 days			Previous Danish Classification up to 1996: 000000-99960; NOMESCO classification after 1996: KA-KQ, KX, KY
Pregnancy within previous 90 days	630-680	O00-O99	

Table S2 *International Classification of Diseases* (ICD) codes used for diseases included in the Charlson Comorbidity Index

	Disease	ICD-8	ICD-10	Score
1	Myocardial infarction	410	I21; I22; I23	1
2	Congestive heart failure	42709; 42710; 42711; 42719; 42899; 78249	I50; I11.0; I13.0; I13.2	1
3	Peripheral vascular disease	440; 441; 442; 443; 444; 445	I70; I71; I72; I73; I74; I77	1
4	Cerebrovascular disease	430-438	I60-I69; G45; G46	1
5	Dementia	29009-29019; 29309	F00-F03; F05.1; G30	1
6	Chronic pulmonary disease	490-493; 515-518	J40-J47; J60-J67; J68.4; J70.1; J70.3; J84.1; J92.0; J96.1; J98.2; J98.3	1
7	Connective tissue disease	712; 716; 734; 446; 13599	M05; M06; M08; M09;M30;M31; M32; M33; M34; M35; M36; D86	1
8	Ulcer disease	53091; 53098; 531-534	K22.1; K25-K28	1
9	Mild liver disease	571; 57301; 57304	B18; K70.0-K70.3; K70.9; K71; K73; K74; K76.0	1
10	Diabetes type 1<br>Diabetes type 2	24900; 24906; 24907; 24909<br>25000; 25006; 25007; 25009	E10.0, E10.1; E10.9<br>E11.0; E11.1; E11.9	1
11	Hemiplegia	344	G81; G82	2
12	Moderate to severe renal disease	403; 404; 580-583; 584; 59009; 59319; 75310-75319; 792	I12; I13; N00-N05; N07; N11; N14; N17-N19; Q61	2
13	Diabetes with end organ damage<br>Type 1<br>Type 2	24901-24905; 24908<br>25001-25005; 25008	E10.2-E10.8<br>E11.2-E11.8	2
14	Any tumor	140-194	C00-C75	2
15	Leukemia	204-207	C91-C95	2
16	Lymphoma	200-203; 27559	C81-C85; C88; C90; C96	2

Table S2 (*Continued*)

	Disease	ICD-8	ICD-10	Score
17	Moderate to severe liver disease	07000; 07002; 07004; 07006; 07008; 57300; 45600-45609	B15.0; B16.0; B16.2; B19.0; K70.4; K72; K76.6; I85	3
18	Metastatic solid tumor	195-198; 199	C76-C80	6
19	AIDS	07983	B21-B24	6

Table S3 Number (%) of cases of deep venous thrombosis, pulmonary embolism, provoked VTE, unprovoked VTE, splanchnic venous thrombosis (1977–2016), cerebral venous thrombosis, and retinal venous thrombosis (1994–2016), by year of diagnosis

	Deep venous thrombosis	Pulmonary embolism	Provoked VTE	Unprovoked VTE	Splanchnic venous thrombosis	Cerebral venous thrombosis	Retinal venous thrombosis
Year of diagnosis							
1977	1,757 (1.7)	2,389 (2.8)	1,470 (2.1)	2,676 (2.3)	105 (2.6)	NA	NA
1978	1,769 (1.7)	2,501 (3.0)	1,642 (2.3)	2,628 (2.3)	84 (2.1)	NA	NA
1979	1,748 (1.7)	2,456 (2.9)	1,738 (2.5)	2,466 (2.1)	91 (2.3)	NA	NA
1980	1,710 (1.7)	2,199 (2.6)	1,541 (2.2)	2,368 (2.0)	89 (2.2)	NA	NA
1981	1,871 (1.8)	2,196 (2.6)	1,602 (2.3)	2,465 (2.1)	84 (2.1)	NA	NA
1982	1,871 (1.8)	2,178 (2.6)	1,711 (2.4)	2,338 (2.0)	71 (1.8)	NA	NA
1983	1,897 (1.9)	2,172 (2.6)	1,667 (2.4)	2,402 (2.1)	76 (1.9)	NA	NA
1984	1,907 (1.9)	2,064 (2.5)	1,731 (2.5)	2,240 (1.9)	79 (2.0)	NA	NA
1985	1,895 (1.9)	1,980 (2.4)	1,622 (2.3)	2,253 (1.9)	90 (2.3)	NA	NA
1986	1,816 (1.8)	1,769 (2.1)	1,486 (2.1)	2,099 (1.8)	97 (2.4)	NA	NA
1987	1,638 (1.6)	1,631 (1.9)	1,357 (1.9)	1,912 (1.6)	87 (2.2)	NA	NA
1988	1,640 (1.6)	1,562 (1.9)	1,339 (1.9)	1,863 (1.6)	54 (1.4)	NA	NA
1989	1,510 (1.5)	1,437 (1.7)	1,215 (1.7)	1,732 (1.5)	81 (2.0)	NA	NA
1990	1,546 (1.5)	1,234 (1.5)	1,115 (1.6)	1,665 (1.4)	91 (2.3)	NA	NA
1991	1,446 (1.4)	1,208 (1.4)	1,058 (1.5)	1,596 (1.4)	71 (1.8)	NA	NA
1992	1,522 (1.5)	1,153 (1.4)	1,013 (1.4)	1,662 (1.4)	58 (1.5)	NA	NA
1993	1,560 (1.5)	1,155 (1.4)	1,040 (1.5)	1,675 (1.4)	66 (1.7)	NA	NA
1994	2,326 (2.3)	1,303 (1.5)	1,353 (1.9)	2,276 (2.0)	52 (1.3)	27 (2.4)	472 (3.0)
1995	2,278 (2.2)	1,378 (1.6)	1,338 (1.9)	2,318 (2.0)	36 (0.9)	31 (2.8)	507 (3.2)
1996	2,324 (2.3)	1,310 (1.6)	1,123 (1.6)	2,511 (2.2)	46 (1.2)	22 (2.0)	499 (3.2)
1997	2,508 (2.5)	1,346 (1.6)	1,127 (1.6)	2,727 (2.3)	47 (1.2)	33 (3.0)	512 (3.3)
1998	2,456 (2.4)	1,408 (1.7)	1,185 (1.7)	2,679 (2.3)	43 (1.1)	31 (2.8)	479 (3.0)
1999	2,588 (2.5)	1,499 (1.8)	1,250 (1.8)	2,837 (2.4)	47 (1.2)	34 (3.0)	488 (3.1)
2000	2,671 (2.6)	1,517 (1.8)	1,303 (1.9)	2,885 (2.5)	51 (1.3)	29 (2.6)	524 (3.3)
2001	2,732 (2.7)	1,598 (1.9)	1,360 (1.9)	2,970 (2.6)	61 (1.5)	20 (1.8)	597 (3.8)
2002	3,047 (3.0)	1,738 (2.1)	1,525 (2.2)	3,260 (2.8)	74 (1.9)	39 (3.5)	544 (3.5)
2003	3,086 (3.0)	1,772 (2.1)	1,679 (2.4)	3,179 (2.7)	85 (2.1)	34 (3.0)	533 (3.4)
2004	3,240 (3.2)	1,919 (2.3)	1,759 (2.5)	3,400 (2.9)	89 (2.2)	62 (5.5)	643 (4.1)
2005	3,442 (3.4)	1,897 (2.3)	1,798 (2.6)	3,541 (3.1)	83 (2.1)	45 (4.0)	598 (3.8)
2006	3,697 (3.6)	2,130 (2.5)	2,039 (2.9)	3,788 (3.3)	99 (2.5)	59 (5.3)	583 (3.7)
2007	3,815 (3.7)	2,275 (2.7)	2,141 (3.1)	3,949 (3.4)	123 (3.1)	52 (4.7)	570 (3.6)
2008	3,515 (3.4)	2,310 (2.7)	2,090 (3.0)	3,735 (3.2)	126 (3.2)	63 (5.6)	632 (4.0)

(*Continued*)

Table S3 (*Continued*)

	Deep venous thrombosis	Pulmonary embolism	Provoked VTE	Unprovoked VTE	Splanchnic venous thrombosis	Cerebral venous thrombosis	Retinal venous thrombosis
2009	3,713 (3.6)	2,543 (3.0)	2,407 (3.4)	3,849 (3.3)	149 (3.8)	53 (4.7)	619 (3.9)
2010	3,635 (3.6)	2,779 (3.3)	2,526 (3.6)	3,888 (3.3)	155 (3.9)	57 (5.1)	659 (4.2)
2011	3,424 (3.4)	2,944 (3.5)	2,462 (3.5)	3,906 (3.4)	160 (4.0)	57 (5.1)	894 (5.7)
2012	3,794 (3.7)	3,347 (4.0)	2,860 (4.1)	4,281 (3..7)	195 (4.9)	55 (4.9)	1,061 (6.8)
2013	3,858 (3.8)	3,642 (4.3)	2,939 (4.2)	4,561 (3.9)	210 (5.3)	63 (5.6)	1,050 (6.7)
2014	3,531 (3.5)	4,020 (4.8)	3,047 (4.4)	4,504 (3.9)	229 (5.8)	67 (6.0)	1,069 (6.8)
2015	3,482 (3.4)	4,136 (4.9)	3,149 (4.5)	4,469 (3.9)	226 (5.7)	95 (8.5)	1,079 (6.9)
2016	3,630 (3.6)	3,985 (4.7)	3,101 (4.4)	4,514 (3.9)	212 (5.3)	90 (8.1)	1,094 (7.0)

Abbreviations: NA, not applicable; VTE, venous thromboembolism.

Table S4 Number of cases (%) of deep venous thrombosis, pulmonary embolism, provoked VTE, unprovoked VTE, splanchnic venous thrombosis (1977–2016), cerebral venous thrombosis, and retinal venous thrombosis (1994–2016), by month of diagnosis

	Deep venous thrombosis	Pulmonary embolism	Provoked VTE	Unprovoked VTE	Splanchnic venous thrombosis	Cerebral venous thrombosis	Retinal venous thrombosis
Month of diagnosis[a]							
January	9,216 (9.2)	7,945 (9.6)	6,179 (9.0)	10,982 (9.6)	310 (7.9)	94 (8.5)	1,342 (8.7)
February	8,732 (8.7)	7,658 (9.2)	6,177 (9.0)	10,213 (8.9)	296 (7.6)	79 (7.1)	1,340 (8.7)
March	8,840 (8.8)	7,184 (8.7)	6,002 (8.7)	10,022 (8.8)	308 (7.9)	97 (8.8)	1,306 (8.4)
April	8,303 (8.3)	6,469 (7.8)	5,565 (8.1)	9,207 (8.0)	321 (8.2)	91 (8.3)	1,160 (7.5)
May	8,168 (8.1)	6,295 (7.6)	5,366 (7.8)	9,097 (7.9)	329 (8.4)	70 (6.3)	1,260 (8.1)
June	8,289 (8.3)	6,332 (7.6)	5,644 (8.2)	8,977 (7.8)	316 (8.1)	93 (8.4)	1,364 (8.8)
July	7,731 (7.7)	6,168 (7.4)	5,115 (7.4)	8,784 (7.7)	295 (7.5)	89 (8.1)	1,117 (7.2)
August	8,220 (8.2)	6,580 (7.9)	5,337 (7.7)	9,463 (8.3)	353 (9.0)	81 (7.4)	1,206 (7.8)
September	8,213 (8.2)	6,999 (8.4)	5,745 (8.3)	9,467 (8.3)	341 (8.7)	111 (10.1)	1,278 (8.3)
October	8,386 (8.3)	6,851 (8.3)	5,898 (8.6)	9,339 (8.2)	341 (8.7)	99 (9.0)	1,394 (9.0)
November	8,411 (8.4)	7,490 (9.0)	6,155 (8.9)	9,746 (8.5)	357 (9.1)	125 (11.3)	1,457 (9.4)
December	7,944 (7.9)	6,957 (8.4)	5,762 (8.4)	9,139 (8.0)	346 (8.9)	75 (6.8)	1,264 (8.2)

Abbreviation: VTE, venous thromboembolism.
[a]Cases were adjusted for the length of the month and rounded to the nearest integer.

Table S5 Number (%) of deaths within 90 days following a deep venous thrombosis, pulmonary embolism, provoked VTE, unprovoked VTE, splanchnic venous thrombosis (1977–2016), cerebral venous thrombosis, and retinal venous thrombosis (1994–2016), by year of diagnosis

	Deep venous thrombosis	Pulmonary embolism	Provoked VTE	Unprovoked VTE	Splanchnic venous thrombosis	Cerebral venous thrombosis	Retinal venous thrombosis
Year of diagnosis							
1977	94 (1.4)	1,286 (4.5)	570 (3.1)	7,718 (4.9)	69 (4.1)	NA	NA
1978	110 (1.7)	1,440 (5.0)	697 (3.8)	8,350 (5.1)	59 (3.5)	NA	NA
1979	118 (1.8)	1,448 (5.1)	756 (4.1)	7,849 (4.9)	75 (4.4)	NA	NA
1980	112 (1.7)	1,261 (4.4)	656 (3.6)	7,675 (4.3)	76 (4.5)	NA	NA
1981	132 (2.0)	1,261 (4.4)	666 (3.6)	7,902 (4.4)	63 (3.7)	NA	NA
1982	132 (2.0)	1,270 (4.4)	694 (3.8)	7,674 (4.2)	54 (3.2)	NA	NA
1983	99 (1.5)	1,200 (4.2)	689 (3.7)	8,051 (3.7)	53 (3.1)	NA	NA
1984	128 (2.0)	1,190 (4.2)	699 (3.8)	7,472 (3.7)	59 (3.5)	NA	NA
1985	107 (1.6)	1,106 (3.9)	633 (3.4)	7,461 (3.5)	67 (4.0)	NA	NA
1986	127 (1.9)	985 (3.4)	587 (3.2)	7,076 (3.1)	71 (4.2)	NA	NA
1987	115 (1.8)	868 (3.0)	496 (2.7)	6,498 (2.9)	64 (3.8)	NA	NA
1988	122 (1.9)	842 (2.9)	486 (2.6)	6,295 (2.9)	33 (1.9)	NA	NA
1989	95 (1.4)	767 (2.7)	461 (2.5)	5,923 (2.4)	46 (2.7)	NA	NA
1990	110 (1.7)	629 (2.2)	381 (2.1)	5,710 (2.1)	71 (4.2)	NA	NA
1991	101 (1.5)	526 (1.8)	342 (1.9)	5,663 (1.7)	49 (2.9)	NA	NA
1992	91 (1.4)	503 (1.8)	302 (1.6)	5,800 (1.7)	33 (1.9)	NA	NA
1993	102 (1.6)	459 (1.6)	273 (1.5)	5,847 (1.7)	39 (2.3)	NA	NA
1994	133 (2.0)	545 (1.9)	346 (1.9)	8,068 (2.0)	21 (1.2)	5 (6.7)	5 (4.1)
1995	140 (2.1)	508 (1.8)	338 (1.8)	8,277 (1.9)	<5	<5	9 (7.3)
1996	138 (2.1)	426 (1.5)	246 (1.3)	9,226 (1.9)	13 (0.8)	<5	<5
1997	168 (2.6)	421 (1.5)	249 (1.4)	9,807 (2.0)	13 (0.8)	5 (6.7)	<5
1998	128 (2.0)	407 (1.4)	240 (1.3)	9,992 (1.8)	8 (0.5)	<5	<5
1999	182 (2.8)	410 (1.4)	264 (1.4)	10,491 (2.0)	20 (1.2)	<5	5 (4.1)
2000	186 (2.8)	403 (1.4)	279 (1.5)	10,611 (1.0)	17 (1.0)	<5	<5
2001	191 (2.9)	390 (1.4)	297 (1.6)	11,061 (1.7)	21 (1.2)	<5	5 (4.1)
2002	215 (3.3)	422 (1.5)	309 (1.7)	12,013 (2.0)	23 (1.4)	<5	7 (5.7)
2003	218 (3.3)	441 (1.5)	344 (1.9)	11,964 (1.9)	28 (1.7)	<5	<5
2004	243 (3.7)	426 (1.5)	337 (1.8)	12,582 (2.0)	31 (1.8)	8 (10.7)	9 (7.3)
2005	254 (3.9)	411 (1.4)	349 (1.9)	13,261 (1.9)	27 (1.6)	<5	<5
2006	253 (3.9)	449 (1.6)	400 (2.2)	14,248 (1.8)	30 (1.8)	<5	6 (4.9)
2007	246 (3.8)	492 (1.7)	412 (2.2)	14,984 (2.0)	43 (2.5)	<5	<5
2008	265 (4.0)	494 (1.7)	456 (2.5)	14,262 (1.8)	30 (1.8)	<5	9 (7.3)
2009	234 (3.6)	533 (1.9)	448 (2.4)	14,393 (1.9)	38 (2.2)	<5	5 (4.1)
2010	205 (3.1)	574 (2.0)	490 (2.7)	14,675 (1.7)	46 (2.7)	<5	5 (4.1)
2011	206 (3.1)	565 (2.0)	481 (2.6)	14,947 (1.7)	39 (2.3)	<5	8 (6.5)
2012	229 (3.5)	601 (2.1)	529 (2.9)	16,088 (1.8)	39 (2.3)	<5	<5
2013	225 (3.4)	616 (2.2)	509 (2.8)	17,431 (2.0)	59 (3.5)	5 (6.7)	7 (5.7)
2014	193 (2.9)	651 (2.3)	561 (3.0)	17,124 (1.7)	62 (3.7)	<5	5 (4.1)
2015	211 (3.2)	704 (2.5)	583 (3.2)	16,993 (2.0)	56 (3.3)	<5	8 (6.5)
2016	202 (3.1)	630 (2.2)	574 (3.1)	17,540 (1.5)	46 (2.7)	5 (6.7)	7 (5.7)

Abbreviations: NA, not applicable; VTE, venous thromboembolism.

Table S6 Number (%) of deaths within 90 days following a deep venous thrombosis, pulmonary embolism, provoked VTE, unprovoked VTE, splanchnic venous thrombosis (1977–2016), cerebral venous thrombosis, and retinal venous thrombosis (1994–2016), by month of diagnosis

	Deep venous thrombosis	Pulmonary embolism	Provoked VTE	Unprovoked VTE	Splanchnic venous thrombosis	Cerebral venous thrombosis	Retinal venous thrombosis
Month of diagnosis[a]							
January	589 (9.1)	2,667 (9.5)	1,585 (8.7)	1,671 (10.2)	133 (7.9)	<5	13 (10.3)
February	599 (9.3)	2,662 (9.5)	1,657 (9.1)	1,605 (9.7)	121 (7.3)	7 (10.0)	14 (11.3)
March	524 (8.1)	2,545 (9.0)	1,546 (8.5)	1,522 (9.2)	134 (8.0)	9 (11.8)	6 (4.8)
April	550 (8.5)	2,276 (8.1)	1,492 (8.2)	1,334 (8.1)	138 (8.3)	6 (8.1)	12 (9.9)
May	529 (8.2)	2,239 (8.0)	1,454 (8.0)	1,315 (8.0)	170 (10.2)	6 (7.8)	6 (4.8)
June	529 (8.2)	2,243 (8.0)	1,499 (8.2)	1,273 (7.7)	120 (7.2)	5 (6.8)	12 (9.9)
July	491 (7.6)	2,163 (7.7)	1,401 (7.7)	1,252 (7.6)	147 (8.8)	5 (6.5)	11 (8.7)
August	491 (7.6)	2,185 (7.8)	1,426 (7.8)	1,249 (7.6)	148 (8.9)	3 (3.9)	7 (5.6)
September	520 (8.0)	2,171 (7.7)	1,496 (8.2)	1,195 (7.3)	134 (8.0)	6 (8.1)	6 (4.9)
October	513 (7.9)	2,237 (7.9)	1,480 (8.1)	1,271 (7.7)	122 (7.3)	7 (9.2)	10 (7.9)
November	571 (8.8)	2,352 (8.4)	1,546 (8.5)	1,377 (8.4)	151 (9.0)	6 (8.1)	18 (14.8)
December	566 (8.7)	2,425 (8.6)	1,594 (8.8)	1,397 (8.5)	151 (9.0)	11 (14.4)	9 (7.2)

Abbreviation: VTE, venous thromboembolism.
[a]Cases were adjusted for the length of the month and rounded to the nearest integer.

Table S7 Peak-to-trough ratios (95% confidence intervals) of summarized monthly cases during 1977–2016 (deep venous thrombosis, pulmonary embolism, provoked VTE, unprovoked VTE, and splanchnic venous thrombosis) and during 1994–2016 (cerebral venous thrombosis and retinal venous thrombosis) using a log-linear Poisson regression model

Deep venous thrombosis	Pulmonary embolism	Provoked VTE	Unprovoked VTE	Splanchnic venous thrombosis	Cerebral venous thrombosis	Retinal venous thrombosis
1.11 (1.09–1.13)	1.23 (1.21–1.26)	1.17 (1.15–1.20)	1.15 (1.14–1.17)	1.11 (1.02–1.22)	1.23 (1.04–1.46)	1.13 (1.08–1.18)
